# Haematopoietic Stem Cell Transplantation for Chronic Granulomatous Disease

**DOI:** 10.3390/jcm12186083

**Published:** 2023-09-20

**Authors:** M. A. Slatter, A. R. Gennery

**Affiliations:** 1 Translational and Clinical Research Institute, Newcastle University, Newcastle upon Tyne NE2 4HH, UK; mary.slatter@nhs.net; 2 Paediatric Stem Cell Transplant Unit, Great North Children’s Hospital, Newcastle upon Tyne NE1 4LP, UK

**Keywords:** chronic granulomatous disease, haematopoietic stem cell transplantation, T-lymphocyte depletion, treosulfan

## Abstract

Chronic granulomatous disease (CGD) is an inborn error of immunity due to defects in the transport or function of subunits of nicotinamide adenine dinucleotide phosphate oxidase, the enzyme that generates the phagocyte respiratory burst responsible for intracellular killing of engulfed micro-organisms. Patients present with infectious or inflammatory complications. Common bacterial pathogens include *Staphylococcus aureus* and *Burkholderia cepacia* complex. Fungal pathogens include *Aspergillus* species, particularly *Aspergillus fumigatus*. Inflammatory complications most commonly manifest as inflammatory bowel disease or lung disease. Granulomata are the distinguishing histological feature. Haematopoietic stem cell transplantation (HSCT) was first considered for CGD in the early 1970’s. Since then, refinements in transplant technique, donor selection, conditioning regimens, and graft engineering have widened the option of HSCT to most patients with CGD. This review charts the progress made in HSCT for CGD.

## 1. Introduction

Chronic granulomatous disease (CGD) is a rare inborn error of immunity caused by a defective phagocyte oxidative burst. Patients are susceptible to infection, particularly with catalase-positive micro-organisms. Conventionally, treatment uses antimicrobial prophylaxis to prevent infection. The majority of patients also experience inflammatory complications, particularly affecting the lung and gut, for which immunosuppression is required. Haematopoietic stem cell transplantation (HSCT) is successful in curing the disease. For X-linked disease, gene therapy has also demonstrated some success in clinical trials. This article reviews the results of HSCT for CGD.

## 2. Pathophysiology and Clinical Outcome

First described in 1954 in children who experienced recurrent infections associated with elevated serum gamma globulin levels [1], the role of phagocytes in the disease process was subsequently recognized [2]. To date, six genetic causes have been identified, with defects in *CYBB*, being the most common in outbred populations and causing X-linked disease, and five genes inherited in an autosomal recessive manner, namely *CYBA*, *CYBC1*, *NCF1*, *NCF2*, and *NCF4* [3]. The genes encode transport or functional subunits of nicotinamide adenine dinucleotide phosphate oxidase, the enzyme that generates the phagocyte respiratory burst responsible for the intracellular killing of engulfed micro-organisms. Patients principally present with infectious or inflammatory complications. Common bacterial pathogens comprise *Staphylococcus aureus* and *Burkholderia cepacia* complex (which is distinguishing for CGD, and pathognomic). *Serratia marsescens*, *Nocardia* species, and mycobacterial species are well described. Fungal pathogens include *Aspergillus* species, particularly *Aspergillus fumigatus*, and are significant pathogens, which are the most common cause of mortality [4,5]. Comparatively few pathogens are implicated in the majority of infections.

Suppurative adenitis, pneumonia, and deep lung, liver, and splenic abscesses are the most frequent infectious presentations [6,7], and are often associated with significant inflammation. Inflammatory complications most commonly manifest as inflammatory bowel disease, which may be mistaken for Crohn’s disease. Inflammatory strictures may cause oesophageal, pyloric, or small bowel obstruction [6,7]. Inflammatory lung disease is also a significant manifestation. Granulomata are the distinguishing histological feature.

Mortality from the disease was around 50% by the end of the second decade of life, until the introduction of azoles as antifungal prophylaxis, according to one French study [8]. Following the introduction of azole prophylaxis, lifespan improved, but mortality remained at 50% by the mid to end of the third decade [9]. Furthermore, patients continued to experience significant infectious complications following diagnosis [6,9] and there is no effective treatment to prevent inflammatory complications. Immunosuppressive treatment of inflammation is associated with an increased risk of infection.

## 3. Haematopoietic Stem Cell Transplantation for Chronic Granulomatous Disease—Historical Perspective

The first therapeutic HSCT procedures were pioneered in 1968 for patients with inborn errors of immunity (for a boy with Wiskott Aldrich syndrome, and 2 infants with severe combined immunodeficiency [10,11,12]). The first reported stem cell transplant for a patient with CGD, from the Westminster Children’s Hospital, London, was published in 1977—a 2-year-old boy, conditioned with cyclophosphamide and receiving marrow from a female unrelated donor, in 1973. Although the patient eventually lost the graft, there was initial clinical improvement [13]. Six further patients were reported with mixed results—two died and one other rejected the graft [14]. However, the principle that the disease could be cured by permanent replacement of the stem cell compartment by a matched healthy donor had been established.

## 4. Haematopoietic Stem Cell Transplantation for Chronic Granulomatous Disease—The Modern Era

A series of 10 patients was reported from the National Institutes of Health (NIH), Bethesda, USA in 2001 [15]. Most had X-linked disease, and all experienced several episodes of life-threatening infection. All received stem cells from an HLA-identical sibling, which were depleted of T-lymphocytes and enriched for CD34^+^ stem cells using an immunomagnetic-bead selection system. Conditioning was with cyclophosphamide (60 mg/kg, days −7 and −6 before transplantation), fludarabine (25 mg/m^2^, days −5 to −1), and antithymocyte globulin (40 mg/kg, days −5 to −2). Patients received between 1–5 aliquots of T-lymphocytes (1.0 × 10^5^ CD3^+^ cells/kg) following transplantation. Despite this reduced-intensity conditioning regimen and use of a T-lymphocyte depleted product, six patients achieved full donor chimerism. Two had mixed chimerism that was curative. There was one primary graft failure and one rejection. Three patients had grade II-IV acute graft versus host disease (aGvHD), whilst two had chronic (c)GvHD. There were 3 deaths, with overall survival (OS) of 70%.

A multi-center European study looked at the outcome of 27 transplant patients from 1985–2000 [16]. All patients had experienced at least 1 invasive infectious episode, and all were categorized into 1 of 3 groups at the time of transplant:- high risk (culture-proven, therapy-refractory, life-threatening infections *n* = 9), medium risk (without overt infection but signs of active ongoing inflammation (colitis) or organ sequelae, due to chronic inflammation, such as pulmonary restriction *n* = 7), and low risk with no active infection or inflammation (*n* = 11). Most had X-linked disease (one was an X-linked CGD carrier with extreme lyonization), 2 had autosomal recessive CGD, and 2 were not characterized. Twenty-five patients received sibling HLA-identical transplants (five of whom were heterozygous carriers). Only 2 patients in the low-risk category received an unrelated donor HLA-identical graft. Most patients received a busulphan-based myeloablative conditioning regimen, predominantly combined with cyclophosphamide. Twenty-two of twenty-three patients who received an HLA-identical unmodified stem cell developed stable full donor chimerism after myeloablative conditioning. The remaining patient died prior to engraftment. Complete donor-derived chimerism was achieved in 2 of 4 patients who received an HLA identical graft following reduced-intensity conditioning whilst the remaining 2 patients had graft failure. In the high-risk group, 3 of 9 patients experienced grade III-IV aGvHD, 5 of 9 survived, 1 with autologous reconstitution. In the medium-risk group, 3 of 7 patients developed aGvHD, and all survived. In the low-risk group, 1 of 11 patients developed aGvHD, and all survived. From the whole cohort, there were 4 deaths (OS 85%), with all deaths occurring in the high-risk group. One survivor had autologous reconstitution. This study confirmed the utility of HSCT to cure CGD and emphasized the increased risk of the procedure in patients with inflammatory disease, and, particularly, active infection at time of transplant.

A single United Kingdom (UK) center study of 20 patients, 19 with X-linked disease, reported on outcomes of using a matched sibling versus an unrelated donor between 1998–2007 [17]. Fourteen had experienced at least one invasive infection prior to HSCT. Ten experienced active inflammatory disease whilst two demonstrated active fungal infection at the time of transplantation. Ten patients received a T-lymphocyte replete stem cell product from an HLA-matched sibling (1 cord blood), 8 received 10/10, and 2 received 9/10 HLA-identical unrelated donor stem cells. For pre-transplant conditioning, 16 received busulphan (16 mg/kg) and cyclophosphamide (200 mg/kg) myeloablative conditioning, with alemtuzumab (1 mg/kg) for non-sibling donor transplants. Two patients, one with active fungal infection and one who demonstrated restrictive pulmonary disease, received fludarabine150 mg/m^2^, melphalan 140 mg/m^2^, and alemtuzumab 1 mg/kg. One adult patient received alemtuzumab 2 mg/kg, fludarabine 150 mg/m^2^ and busulphan 8 mg/kg, whilst the other was given CAMPATH 1-G (100 mg total dose), busulphan 16 mg/kg and melphalan 140 mg/m^2^. All patients received GvHD prophylaxis-cyclosporine in all, 12 with additional methotrexate and 4 with prednisolone. Two patients died, one from each group, giving an OS of 90%. The oxidative burst was normal or >70% of neutrophils in 15 patients and remained >30% in the other 3 surviving patients. Nine patients experienced aGvHD:- in two it was significant, both with pre-existing inflammation. In all survivors, the disease was cured, with a sustained phagocyte oxidative burst above that required for normal host defence. There was a complete lack of infection documented beyond the transplant period and antimicrobial prophylaxis was discontinued. Serious infection (pneumonia, suppurative adenitis, liver or lung abscess, septicaemia, or osteomyelitis) had been documented in a previous study of non-transplanted patients every 3.9 patient follow up years [6]. No such infections were documented in 80.5 patient follow up years in this post-transplant series. Furthermore, there was resolution of colitis and other inflammation, and catch up growth was demonstrated in many patients. This study demonstrated similar outcomes when using matched sibling or unrelated donors and demonstrated that the procedure was curative with lack of infectious episodes following transplantation, resolution of inflammation, and re-establishment of normal growth.

A follow up study of the UK CGD pediatric cohort compared outcomes of a cohort of 30 patients transplanted at ≤16 years of age (median follow-up after transplantation 4.42 years [range, 2.25–9.00 years]), with an age-matched cohort of 32 non-transplanted patients [18]. The study documented that non-transplanted children had more infections and admissions to the hospital than children followed post-transplant, and that height and body mass index were significantly higher in the post-transplant group. Survival in each cohort was 90%. This study demonstrated that survival with conventional prophylactic treatment in the modern era, at least in the pediatric age range, is very good, and comparable to patients undergoing HSCT, although physical outcomes are better in the transplanted group. Long term follow-up data on the transplanted cohort are required, but long-term data of transplant outcomes for other inborn errors of immunity are very good, and similar outcomes would be expected for patients transplanted for CGD. A study of the UK CGD adult non-transplanted cohort, comprising 53 patients demonstrated significant morbidity due to inflammatory and infectious complications [19]. The survival at median age of follow-up (30 years) was 100%, the survival probability for all patients was 94.7%, 88%, 79%, and 59% at ages 36, 38, 44, and 50 years, respectively.

A recent Inborn Errors Working Party (IEWP) of the European Blood and Marrow Transplantation (EBMT) society study performed a retrospective multicenter study of 712 patients with CGD, from 101 centers, who underwent HSCT between March 1993–December 2018. The cohort of 712 patients included 635 children (aged < 18 years) and 77 adults [20]. The majority (620) of patients were transplanted after 2005. The most common conditioning regimen employed was busulfan and fludarabine (45.5%). Busulfan and cyclophosphamide conditioning was used in 15.9%. Treosulfan-based conditioning with fludarabine was used in 12.5% and 8.1% with the addition of thiotepa, reflecting the IEWP guidelines in effect during the period of study [21]. Patients received stem cells from matched related or unrelated donors in 547 of the 681 cases (80%), and 20% received grafts from mismatched donors. Median follow-up was 45 months (range 18–83 months). The Kaplan–Meier estimate of OS at 3 years post-transplant was 85.7% and of event-free survival (EFS) at 3 years post-transplant was 75.8%. The cumulative incidence of grade II-IV aGvHD was 20.1%. The cumulative incidence of grade III-IV aGVHD was 9%. The cumulative incidence of cGVHD at 3 years was 17.8% and of extensive cGVHD at 3 years was 6.2%. Older age at transplant was associated with reduced survival and an increased incidence of cGVHD. Despite that, OS and EFS at 3 years for patients transplanted at ≥18 years were 76% and 69%, respectively. The selection of conditioning regimen did not affect OS or EFS. The study conclusions were that transplantation should be strongly considered at a younger age, particularly if a well-matched donor was available. Good results were possible with older patients, however.

A Japanese study reported the outcome of a national cohort of 91 patients transplanted between 1992–2013 [22]. The median age at transplant was 11 years (range 0–39 years). A total of 64 patients had proven X-linked disease, 13 had autosomal recessive disease, and 14 were genetically undetermined. A total of 27 and 36 patients received a transplant from an HLA-matched sibling or unrelated donor, respectively. The remaining patients received stem cells from mismatched related (8), unrelated (17), and related haplo-identical donors (3). Myeloablative conditioning, including radiation-based regimens, was employed in 24 patients—the remainder received reduced intensity conditioning. A total of 70 patients were alive at a median follow-up of 39 (4–230) months. Three-year OS and EFS were 73.7% and 67.6%, respectively. Twenty-one patients died mainly from transplant-related mortality. The cumulative incidence of grade II to IV aGVHD was 27.2%. The cumulative incidence of extensive cGVHD was 17.9%.

The Primary Immune Deficiency Treatment Consortium compared 151 conventionally treated patients with 240 transplant patients enrolled from North American centers between 2004–2018, in a multi-institutional retrospective and prospective study [23]. Of the transplanted patients, 178 had matched and 62 mismatched donors, including 13 who received a T-lymphocyte depleted stem cell product. Myeloablative conditioning was administered to 78 patients—a variety of myeloablative and reduced intensity regimens were employed. The median follow-up post-transplant was 3.7 years (range 2–6 years). In the transplant group, 3-year OS was 82% and EFS was 69%. Cumulative incidence of grade II-IV aGvHD was 18.4% and of grade III-IV aGvHD was 6.2% (95% CI 3.5–9.9%). Patients who received serotherapy as part of the conditioning regimen had significantly less grade II-IV aGVHD. The design of the study meant it was not possible to calculate survival in the non-transplanted cohort. However, transplanted patients had significantly less infections and inflammatory disease, and none required treatment with corticosteroids.

The pre-transplant pre-morbid condition of the patients impacts the outcome, as demonstrated in the early European study that risk-stratified patients [16]. In that study, active infection was associated with mortality, but whilst inflammatory disease was associated with an increased risk of GvHD, there was no increased mortality. In the large study by Chiesa et al. [20], there was a trend towards reduced survival in patients with chronic inflammatory bowel disease compared with those without colitis (*p* = 0.052). However, a study by Marsh et al. [24] compared a transplant cohort of 49 patients who experienced inflammatory bowel disease associated with CGD with 96 CGD patients without bowel disease. There was no significant difference in engraftment, upper or lower gut aGVHD, or cGVHD between the cohorts. The 5-year OS was equivalent. Differences in outcome between the cohorts may relate to differences in cohort size. In the European study, presence of colitis did not associate with an increased risk of aGvHD or cGVHD, possibly because of the use of in vivo T-lymphocyte depletion with pre-transplant serotherapy. Regardless, patients who experience granulomatous colitis would be expected to benefit from optimization of the immunosuppressive strategy before they proceed to transplantation, as control of the bowel disease will likely reduce the inflammatory load.

## 5. Most Appropriate Pre-Transplant Conditioning Regimen

Whilst early studies used conventional myeloablative conditioning regimens, poor condition of some patients made it difficult for them to tolerate the treatment, and significant toxicities in survivors, such as sinusoidal obstructive syndrome, led the way to develop regimens that were better tolerated, safer, and had less long term adverse effects. Gϋngor and colleagues reported on an international prospective study of 56 patients from 16 centers who received a reduced intensity conditioning regimen composed of high-dose fludarabine (180 mg/m^2^), anti-thymocyte globulin (40 mg/kg) or thymoglobuline (7.5 mg/kg), or low-dose alemtuzumab (<3 mg/kg on days −8 to −6]), and low-dose (50–72% of myeloablative dose) or targeted busulfan administration (with a recommended cumulative area under the curve of 45–65 mg/Lxh) [25]. A total of 13 of the 56 patients were adults ≥18 years at transplant. Forty were defined as high risk, based on infectious or inflammatory history. Further, 21 patients received cells from matched related donors, 25 from HLA-matched, and 10 from 1-antigen HLA-mismatched unrelated donors. The conditioning regimen was well tolerated, with no episodes of sinusoidal obstruction syndrome, interstitial pneumonitis, or mucositis of grade III–IV occurring. With a median follow-up of 21 months (range 13–35 months), OS was 93% and EFS was 89%. HLA-matched related-donor transplants had a better OS and EFS, respectively, than mismatched donor transplants (OS 100% vs. 94%, EFS 95% vs. 89%). Three patients experienced early (*n* = 1) or late (*n* = 2) graft failure. The cumulative incidence of grade III–IV aGVHD was 4% (two of 56) and cGvHD was 7%. Stable (≥90%) myeloid donor chimerism was documented in all surviving patients.

Treosulfan is a water-soluble bi-functional alkylating agent that is a dihydroxy-busulfan derivative. The hydrophilic properties of treosulfan offer less hepatic distribution than busulfan, thus reducing the severity and incidence of hepatic complications, and particularly sinusoidal obstruction syndrome. The lack of hepatic metabolism reduces interaction with concurrent medications, and treosulfan is significantly less neurotoxic than busulfan. Morillo-Gutierrez and colleagues reported a retrospective international study from 16 centres [26]. Seventy patients (median age, 107 months, range 46–232 months) transplanted between 2006—2015 were included. Most had high-risk features. A total of 57 had HLA-matched donors, 12 had HLA-mismatched donors, and 1 received a T-lymphocyte depleted parental haploidentical transplant. The choice of the conditioning regimen was decided by the institution, but with treosulfan as the primary myeloablative agent. Two main groups were patients who received treosulfan, fludarabine, and serotherapy with either antithymocyte globulin or alemtuzumab (46 patients), and 24 patients received other regimens, with 15 patients receiving treosulfan, fludarabine, thiotepa, ATG, or alemtuzumab. No major conditioning-related toxicity was reported. At a median follow-up of 34 months (range 13–102 months), the OS was 91.4%, and EFS was 81.4%. Cumulative incidence of grade III-IV aGVHD was 12%. Nine patients developed cGVHD. Complete myeloid donor chimerism was documented in 80% of surviving patients. Secondary graft failure occurred in 8 (12%) patients.

A study from the NIH reported outcomes of 40 patients who underwent HSCT between 2007–2015 using a reduced intensity conditioning regimen [27]. Forty patients received matched sibling stem cells (4), matched unrelated donor cells (35), and one patient received mismatched unrelated cells. Conditioning consisted of alemtuzumab 1 mg/kg and non-dose adjusted intravenous busulfan 10 mg/kg. Patients who had an unrelated donor were given 300 cGy total body irradiation (TBI) on day -1. Sirolimus was given as GvHD prophylaxis. Mean age at transplant was 16 years (range 4–32 years), 23 (58%) were children <18 years of age. Two patients experienced primary graft failure. At a median follow up of 3.4 years, OS was 82.5% and EFS 80%. Cumulative incidence of grade II-IV aGVHD was 45%, (18 patients), from which there were 6 deaths. Five patients developed cGVHD. Complete myeloid donor chimerism was documented in 84% of surviving patients, and all surviving patients were free of symptoms related to CGD.

The large European study did not find any differences in clinical outcomes between patients who received busulphan or those that received treosulfan [20]. It is likely that traditional survival and event free survival differences between the two regimens are likely to be very small. However, long-term follow up will be required to determine differences in parameters such as fertility rates

## 6. Donor Selection

Although in most studies the majority of patients have received the graft from a matched donor, this is not possible for all patients. Outcomes are acceptable using mismatched donors, but generally have not been as good as for matched donors. In order to prevent GvHD, T-lymphocyte depletion is employed, but this prolongs T-lymphocyte recovery, exposing the patient to an increased infectious risk during the immune reconstitution phase [28]. In recent years, the outcome of transplantation using T-lymphocyte-depleted products from mismatched donors has significantly improved. Two platforms dominate the field currently: immunomagnetic bead in vitro depletion of CD3 TCRαβ^+^ and CD19^+^ lymphocytes, and administration of 2 doses of cyclophosphamide following infusion of a replete inoculum, leading to in vivo depletion of proliferating T-lymphocytes.

A single-center experience of transplantation for CGD documents outcomes of 55 pediatric patients transplanted between 1998–2017 [29]. The 5-year OS was 89% but was 100% for children transplanted at <5 years of age vs. 81% for children transplanted at >5 years. The OS was the same for matched family and unrelated donor transplants. Only four patients received haploidentical transplants after conditioning with fludarabine, treosulfan, thiotepa, anti-thymocyte globulin, and rituximab, but all were successfully treated and were cured with 100% donor chimerism. Three of these did not receive anti-GvHD chemoprophylaxis, and none developed GvHD.

The NIH reported on 7 patients who received a mismatched family donor stem cell source followed by post-transplant cyclophosphamide [30]. Patients were aged 14–26 years. Patients received fludarabine (150 mg/m^2^), busulfan (with measured levels to achieve a total area under the curve 4000–6000 min × μMol/L), cyclophosphamide 29 mg/kg, and TBI (200 cGy) as pre-transplant conditioning, with post-transplant cyclophosphamide (50 mg/kg days 3 and 4) and sirolimus as GvHD prophylaxis. All patients engrafted. All patients had grade II or higher aGvHD; 2 also developed mild cGvHD. There was 71% OS: the 2 deaths were due to steroid-refractory GvHD associated with viral infection, 1 with the associated post-transplant lympho-proliferative disease.

Although there were a number of patients who had received T-lymphocyte depleted stem cell products in the large IEWP series [20], there were too few to make a meaningful comparison. An IEWP study is currently examining this particular question. However, from the limited published data available, it seems that in the modern era, outcomes using mismatched donors and a T-lymphocyte depleted product are similar to those using matched donors.

## 7. X-Linked Chronic Granulomatous Disease—Special Groups

### 7.1. Mcleod Phenotype

A specific group of patients with X-linked CGD for whom HSCT can be particularly difficult is those with the McLeod phenotype. The McLeod phenotype is a neuroacanthocytosis syndrome associated with X-linked CGD. It is caused by large deletions on the X-chromosome, which include *CYBB* and *XK*. *XK* encodes the Kx transmembrane protein, which attaches to the Kell glycoprotein, found on erythyrocytes. The McLeod blood phenotype is uncommon. It is characterized by reduced Kell glycoprotein antigen expression. Patients are at risk of developing an allo-immune response if they have received erythrocyte or granulocyte transfusions. Subsequently, they may acquire brisk haemolysis as an effect of incompatible transfusions delivered as supportive care through the transplantation period. One study describes 8 patients with McLeod phenotype, 4 of whom underwent transplant and 3 of whom survived [31].

### 7.2. X-Linked-Carriers

Female carriers of X-linked CGD harbor only one copy of mutated *CYBB* on the X chromosome, and as a result of lyonisation, have two phagocyte populations. One is gp91^phox^ positive, derived from the normal X-chromosome and the other is gp91^phox^ negative, derived from the X-chromosome carrying mutated *CYBB*. Random X-chromosome inactivation in early fetal development of haematopoietic precursor cells may lead to two unequal populations of circulating phagocytes; those which are able to produce a respiratory burst and subsequently a superoxide and those which cannot. It is now recognized that female carriers may be symptomatic, with inflammatory manifestations and significant fatigue [32,33]. For this reason, carriers of X-linked disease are avoided as potential donors if possible. With extreme lyonisation, the phagocyte respiratory oxidative burst may be so reduced that carriers present with infections that mirror those of male patients with X-linked CGD [34]. Even in early transplant series, X-linked carriers were described [16]. A recently published series documents outcomes of transplantation in 7 symptomatic female carriers aged 1–56 years [35]. The transplant was considered for severe and recurrent infection, colitis, and autoimmunity. All patients engrafted with restoration of the oxidative burst, although two patients succumbed subsequently to transplant-related complications. Surviving patients demonstrated a reduction in infection and inflammatory symptoms and freedom from immunosuppressive therapy.

## 8. Conclusions

Since the early description of the disease as “Fatal granulomatous disease of childhood”, the field has made significant progress. Today, HSCT should be considered standard of care and should be considered early after diagnosis. All genetic forms of CGD respond to transplantation, including the recently described and rare form due to mutations in *CYBC1* [36]. Whilst the ideal conditioning regimen has yet to be determined, reduced toxicity regimens, particularly using treosulfan or targeted busulfan, appear to confer benefit by enabling stable myeloid engraftment with disease correction, but reducing the unwanted toxic effects of chemotherapy. Whilst a fully matched donor may still be considered the best option, alternative mismatched donors may be used successfully with T-lymphocyte-depleted products, although further data are required to clarify the most optimal method of achieving this. Best outcomes are achieved with transplantation at a young age, before permanent organ damage has occurred, but nevertheless, successful outcomes can be realized in adults, and transplantation should not be discounted as a curative option for this population. Early transplant will enable normal growth potential, and abolish the risk of infection and risk of inflammatory complications. Transplanted patients also achieve a normal life quality, in contrast to those who remain on medical treatment [37] and are able to discontinue prophylactic medication. Many have families and lead normal lives. Unanswered questions remain—including optimal age for transplantation, best conditioning regimen, and required level of donor chimerism. Ideally, high level (>80%) donor myeloid chimerism will be achieved, although stable donor myeloid chimerism >15–20% seems adequate in our experience, and we would not advocate re-transplanting a patient judged on chimerism level alone but based on symptom history. Stable recipient T-lymphocyte chimerism >2 years post-HSCT is generally tolerated and does not require treatment.

For some patients, HSCT may not be an option, perhaps because their severe pre-morbid condition precludes transplantation. For a few of these patients, gene therapy may be an option—clinical trials using lentiviral vectors are now available for patients with mutations in *CYBB* (Clinicaltrials NCT02234934) and *NCF1* (Clinicaltrials NCT05207657). Whichever approach is considered, much progress has been made since the first description of the disease, and patients should now expect to lead a normal life.
